# The Haunting of Medical Journals: How Ghostwriting Sold “HRT”

**DOI:** 10.1371/journal.pmed.1000335

**Published:** 2010-09-07

**Authors:** Adriane J. Fugh-Berman

**Affiliations:** Department of Physiology and Biophysics, Georgetown University Medical Center, Washington, D.C., United States of America

## Abstract

Adriane Fugh-Berman examines documents unsealed in recent litigation to investigate how pharmaceutical companies promoted hormone therapy drugs, including the use of medical writing companies to produce ghostwritten manuscripts and place them into medical journals.

**Summary Points**

- Some 1500 documents revealed in litigation provide unprecedented insights into how pharmaceutical companies promote drugs, including the use of vendors to produce ghostwritten manuscripts and place them into medical journals.
- Dozens of ghostwritten reviews and commentaries published in medical journals and supplements were used to promote unproven benefits and downplay harms of menopausal hormone therapy (HT), and to cast raloxifene and other competing therapies in a negative light.
- Specifically, the pharmaceutical company Wyeth used ghostwritten articles to mitigate the perceived risks of breast cancer associated with HT, to defend the unsupported cardiovascular “benefits” of HT, and to promote off-label, unproven uses of HT such as the prevention of dementia, Parkinson's disease, vision problems, and wrinkles.
- Given the growing evidence that ghostwriting has been used to promote HT and other highly promoted drugs, the medical profession must take steps to ensure that prescribers renounce participation in ghostwriting, and to ensure that unscrupulous relationships between industry and academia are avoided rather than courted.

## Introduction

In recent litigation against Wyeth, more than 14,000 plaintiffs brought claims related to the development of breast cancer while taking the menopausal hormone therapy Prempro (conjugated equine estrogens [CEEs] and medroxyprogesterone acetate [MPA]). Some 1500 documents revealed in the litigation provide unprecedented insights into how pharmaceutical companies promote drugs, including the use of vendors to produce ghostwritten manuscripts and place them into medical journals. These documents became public when *PLoS Medicine* and *The New York Times* intervened in the litigation. Both intervenors successfully argued that ghostwriting undermines public health and that documents proving the practice should be unsealed.

In this Policy Forum article, I use these documents, which are available through PLoS at http://www.plosmedicine.org/static/ghostwriting.action or at the Drug Information Document Archive at http://dida.library.ucsf.edu/documents.jsp to show how industry uses ghostwriters to insert marketing messages into articles published in medical journals. As a paid expert witness, I had access to these documents during the litigation but I have received no payment for researching or writing this Policy Forum.

## Hormone Therapy History

In 1942, Premarin (CEE) became the first FDA-approved treatment for hot flashes. Promotional efforts implied that estrogen could preserve youth and health. By the early 1970s, physicians, under the mistaken impression that menopause was an endocrine disease similar to hypothyroidism, were prescribing estrogen to millions of asymptomatic women. In 1975, an eight-fold increase in endometrial cancer was linked to estrogen use, and estrogen sales decreased [1].

After adding a progestin pill to counteract estrogen-induced endometrial cancer, hormone “replacement” therapy (HRT; now properly termed menopausal hormone therapy, or HT) became popular in the 1980s. Through the 1990s, HT was touted to prevent cardiovascular disease, osteoporosis, Alzheimer's disease, colon cancer, tooth loss, and macular degeneration [1]. Prempro, which combined CEE and the progestin Provera (medroxyprogesterone acetate), was approved in the U.S. in 1995. In 1998, the Heart and Estrogen/progestin Replacement Study (HERS), a randomized controlled trial (RCT) in women with cardiovascular disease, found no benefit of HT for preventing cardiovascular events [2]. In 2002, the Women's Health Initiative (WHI), a large RCT in healthy women, demonstrated conclusively that HT failed to prevent cardiovascular disease, increased the risk of breast cancer and stroke, and reduced fracture risk [3],[4]. Later analyses revealed that HT increased the risk of dementia [5] and incontinence [6].

Today, despite definitive scientific data to the contrary, many gynecologists still believe that the benefits of HT outweigh the risks in asymptomatic women [1],[7]–[8]. This non-evidence–based perception may be the result of decades of carefully orchestrated corporate influence on medical literature.

## Publication Planning

Publication planning is the process by which pharmaceutical, biotech, and medical device companies produce and release articles in medical journals and posters at meetings to establish key marketing messages [9],[10]. Some companies employ writers and publication planners, and most hire medical education and communication companies (MECCs) to create publications. Academic physicians are invited by these MECCs to “author” prewritten articles [11],[12]. It is unknown how many academics participate, or how many articles in peer-reviewed medical journals are ghostwritten, but there is concern that the practice may be extensive.

Between 1996 (when Prempro was first marketed) and 2004, Wyeth worked with several MECCs, but most closely with DesignWrite, to promote the Premarin family of products. DesignWrite offers comprehensive services to pharmaceutical companies and has helped to promote topiramate, epoietin alfa, etanercept, and many other drugs [13]. Indeed, according to DesignWrite's website, over 12 years DesignWrite “… planned, created, and/or managed hundreds of advisory boards, a thousand abstracts and posters, 500 clinical papers, over 10,000 speakers' bureau programs, over 200 satellite symposia, 60 international programs, dozens of websites, and a broad array of ancillary printed and electronic materials” [14].

In its communications with Wyeth, DesignWrite noted that “Research shows high clinician reliance on journal articles for credible product information.” In addition to “full-length review articles,” DesignWrite recommended that the publication plan for Premarin products should include mini-reviews, case reports, editorials, letters, and comments [15]. These short pieces could be published quickly, DesignWrite noted, so were an efficient “means of placing important information about the therapeutic profile of an agent into the hands of influential physicians …” [15]. DesignWrite also explained that it would help Wyeth decide what data to present, recruit “authors,” choose journals, create abstracts and posters for medical meetings, and “Position the product appropriately to influence prescribers” [15].

During its work with Wyeth, DesignWrite wrote the first drafts of articles and submitted them to Wyeth. DesignWrite then incorporated Wyeth's comments into a second draft, and sent the company-approved draft to the “author,” whose comments, if any, were incorporated into the third draft. DesignWrite then assisted in submitting the paper to a journal [15]. There is no evidence that authors were paid for authoring articles. Throughout the documents referred to in this Policy Forum, “writer” refers to the ghostwriter, and “author” refers to the person whose name appeared on the published article [16].

Between 1997 and 2003, DesignWrite's output for Wyeth on the Premarin family of products included “over 50 peer-reviewed publications, more than 50 scientific abstracts and posters, journal supplements, internal white papers, slide kits, and symposia…” [17]. Primary publications (articles that report clinical trials) ghostwritten by DesignWrite included four manuscripts on the HOPE trials of low-dose Prempro [18],[19] for which DesignWrite was paid US$25,000 each [20]. Secondary publications (articles that follow clinical trial reports and contain “subsequent analyses, and reviews of the drug and its field of use” [10]) included 20 review articles that DesignWrite was assigned to write in 1997 [21] for $20,000 each [22], a price that later rose to $25,000 [23]. Abstract production cost $4,000. [24] DesignWrite charged $10,000 for editing manuscripts and $2,000 for editing abstracts “written by author or other agency” [24].

As part of its publication planning, Wyeth's Marketing Department convened monthly meetings to discuss publication strategies [25], draft outlines [26],[27], and sometimes adjust the overall publication plan. In 2002, for example, Wyeth management “charged the Publication Committee with increasing the number of positive HRT/Premarin-related publications. They have asked us to publish at least 1 study per month” [28].

## Unregulated Marketing through Medical Journals

It is illegal for pharmaceutical companies to promote a marketed drug for off-label use, i.e., for uses other than those approved by the U.S. Food and Drug Administration (FDA) or equivalent national agencies. Articles in medical journals, newsletters, and magazines, however, are not considered promotional. As an industry article states, “Peer-reviewed publications offer pharma companies shelter from often-stormy regulatory waters. FDA views published articles as protected commercial speech so doesn't regulate their content” [29].

In the absence of data (or in the presence of data adverse to marketing goals), review articles in medical journals are crucial vehicles for encouraging off-label uses, promoting unproven benefits, and for downplaying harms. Narrative reviews summarize and analyze prevailing literature and often offer clinical recommendations [30]. Commentaries and other opinion pieces are also highly valued because they provide clinical direction, and are usually not peer-reviewed. Presentations at medical meetings are important for the same reason [30].

As Table 1 shows, DesignWrite helped to produce numerous ghostwritten reviews and commentaries, including articles designed to promote the off-label use of Prempro for preventing Alzheimer's disease, Parkinson's disease, age-related macular degeneration, and wrinkles. The scope of these articles is summarized in Box 1. The DesignWrite documents avoid discussing off-label marketing, but noted that reviews can “Disseminate messages that fill the gaps not addressed by current studies” [31]. Another document noted that the “Strategic Publications Team” should “Identify data gaps” and “Fill the gap with review papers” [32].

**Table 1:** Examples of ghostwritten reviews and commentaries*.

Mitigating Perceived Risks of Breast Cancer	
**Article**	**Messages From Published Article**
Creasman WT. Is There an Association between Hormone Replacement Therapy and Breast Cancer? J Women’s Health 1998; 7(10)	“In aggregate, these data fail to provide definitive evidence that the use of postmenopausal HRT is associated with an increased incidence of breast cancer.”
Nachtigall LE. Sex Hormone-Binding Globulin and Breast Cancer Risk Primary Care Update for Ob/Gyns 1999; 6 (2):39-45.	“Extensive epidemiologic studies provide conflicting evidence as to whether ERT significantly impacts the risk of breast cancer in postmenopausal women…”“The increase in SHBG promoted by oral ERT, and especially by oral conjugated estrogens, might contribute to the favorable epidemiologic data for this class of estrogens with respect to the breast cancer rate. ”
Eden J. Progestins and breast cancer. Am J Obstet Gynecol. 2003 May;188(5):1123-31.	“… studies have clearly demonstrated that prior or current HRT use results in a paradoxically improved survival for patients with breast cancer.”“…results from epidemiologic studies are inconsistent and mechanistic studies have not provided a physiologic foundation to implicate progestin in the pathogenesis of breast cancer.”
Cefalu T. The Use of Hormone Replacement Therapy in Postmenopausal Women with Type 2 Diabetes. J Women’s Health 2001; 10 (3):241-255	“Although a possible risk has been shown in long-term users, a causal relationship between ERT/HRT and breast cancer remains controversial.”
**Promoting Unproven, Off-Label Uses**	
Fillit, M. The Role of Hormone Replacement Therapy in the Prevention of Alzheimer Disease. Arch Intern Med. 2002;162(17):1934-42.	“At present, most observational evidence, which is supported by neurobiological research findings on the action of estrogen, indicates that ERT/HRT mitigates the degeneration that may lead to AD. The lack of evidence of a role of estrogen in the treatment of AD suggests that ERT/HRT should be initiated as early as possible after menopause, before the onset or the progression of the disease.”
Birge SJ. Practical Strategies for the Diagnosis and Treatment of Alzheimer’s Disease. Clinical Geriatrics 1999 7(4):56-74.	“Estimates of the annual cost of AD per individual range from $34,000 to $47,000, with the annual overall cost to society estimated at $67 billion.”“…effective treatment in the form of disease prevention or delayed expression would significantly decrease the financial burden to both the individual and society. Delaying institutionalization by just one month would reduce that cost by 1.2 billion dollars.”
Shulman L. Is there a Connection Between Estrogen and Parkinson’s Disease? Parkinsonism Relat Disord. 2002;8(5): 289-95	“Increasing evidence suggests that estrogens may protect the nigrostriatal dopaminergic pathway affected in Parkinson’s disease (PD).”
Sherwin BB. Mild Cognitive Impairment: Potential Pharmacological Treatment Options. J Am Geriatr Soc. 2000;48(4):431-41.	“Estrogen, in particular, deserves more attention because its cognitive-enhancing properties, which have been verified by several controlled clinical trials, are complemented by its potential for preventing cardiovascular disease and osteoporosis and for reducing the risk of colorectal cancer and all-cause mortality in postmenopausal women.”
Brincat M, Baron Y, Galea R. Estrogens and the Skin. Climacteric 2005;8(2):110-23.	“Estrogen treatment in postmenopausal women has been repeatedly shown to increase collagen content, dermal thickness, and elasticity. … Physiologic studies on estrogen and wound healing suggest that HRT may play a beneficial role in cutaneous injury repair; however, molecular studies have yet to articulate the mechanisms.”
Snow KK, Seddon JM. Age-Related Eye Diseases: Impact of Hormone Replacement Therapy and Other Risk Factors. Int J Fertil Womens Med. 2000 Sep-Oct;45(5):301-13	“Evidence suggests that among women, long-term exposure to endogenous estrogens or replacement estrogens may reduce the risk of AMD and cataracts. … The potential value of this therapy in reducing visual impairment among women deserves increased attention.”
Freedman, MA.Quality of Life and Menopause: The Role of Estrogen.J Women’s Health 2002;11(8):703-718.	“Less attention has been paid to the menopausal symptoms that can impair the quality of life of menopausal women, such as hot flushes, sleep disorders, sexual dysfunction, and alterations in mood… Evidence supporting the effectiveness of ERT/HRT in the treatment of symptoms affecting quality of life is growing and supports the use of ERT/HRT during menopause.”
Bachman G, Leiblum S. The Impact of Hormones on Menopausal Sexuality: a Literature Review. Menopause 2004;11 (1): 120-130.	“Estrogen deficiency initially accounts for altered bleeding and diminished vaginal lubrication. Continual estrogen loss often leads to numerous signs and symptoms, including changes in the vascular and urogenital systems. Alterations in mood, sleep, and cognitive functioning are common as well. These changes may contribute to lower self-esteem, poorer self-image, and diminished sexual responsiveness and sexual desire.”
Cefalu T. (above)	”The potential in a diabetic population for improved insulin and glucose metabolism, as well as reduced risk of CVD, with the use of ERT/HRT has been shown in several prospective studies.”
Gallagher JC.	“The beneficial effects of estrogen on the prevention of osteoporosis are likely to carry over to improved dental health in women.”
**Competitive Messaging**	
Gallagher JC. Role of Estrogens in the Management of Postmenopausal Bone Loss. Rheum Dis Clin North Am. 2001;27(1):143-62.	“ERT remains the therapy of choice because of its long-term effect on BMD and because estrogen has other favorable systemic benefits in addition to the prevention of osteoporosis.”“Of the oral preparations, the best studied in postmenopausal women has been conjugated equine estrogens ([CEE] Premarin). “
Mosca L. The Role of Hormone Replacement Therapy in the Prevention of Postmenopausal Heart Disease, Arch Intern Med. 2000 Aug 14-28;160(15):2263-72.	“The results of preclinical studies with SERMs suggest smaller cardiovascular effects than those seen with HRT.”
Warren M. A Comparative Review of the Risks and Benefits of Hormone Replacement Therapy Regimens. Am J Obstet Gynecol. 2004 Apr;190(4):1141-67	“Overall, these data indicate that the benefit/risk analysis that was reported in the Women's Health Initiative can be generalized to all postmenopausal hormone replacement therapy products.”
Curtis M. Selective Estrogen Receptor Modulators: A Controversial Approach for Managing Postmenopausal Health. J Women’s Health 1999; 8 (3) : 321-33	“HRT, the current standard of care, has the advantage of long-term epidemiologic data that indicate that the benefits of therapy clearly outweigh the risks. In contrast, the risk:benefit of the emerging SERMs needs to be better defined and evaluated.”“The clinical use of SERMs has yet to demonstrate beneficial effects shown with HRT on all-cause mortality, colon cancer, and central nervous system function (i.e., reduced risk of Alzheimer's disease, improve cognition).”
Curtis MG. Comparative Tolerability of First-Generation Selective Estrogen Receptor Modulators in Breast Cancer Treatment and Prevention. Drug Safety 2001;24(14):1039-53	“At present, each potential adverse event needs to be weighed against potential benefits in the decision to undergo SERM treatment…”“The development of future generations of SERMS that improve upon the current therapies is eagerly anticipated.”
Bachmann GA. Menopausal Vasomotor Symptoms: a Review of Causes, Effects and evidence-Based Treatment, J Reprod Med. 2005 Mar;50(3):155-65.	[Regarding non-pharmacological interventions]: “Although anecdotal reports have suggested that some of these strategies may provide relief, few patients seem to benefit from these interventions.”[Regarding SSRIs]: “the utility of these drugs is restricted by frequent side effects.”[Regarding alternative medicine]: “…no better, or slightly more effective, than placebo.”
Ansbacher R. The Pharmacokinetics and Efficacy of Different Estrogens are Not Equivalent. Am J Obstet Gynecol. 2001 Feb;184(3):255-63	“Generic conjugated estrogens have been manufactured; however, the therapeutic equivalence of these generic products to CEE cannot be ensured…”
No author listed. Generic and Therapeutic Substitution. National Pharmacy Compliance News 2000;4^th^ quarter:2-3.	‘“I've seen quite a bit of confusion regarding the substitutability of certain drugs, most recently between Premarin (conjugated estrogen tablets, USP) and Cenestin (synthetic conjugated estrogens, A),” says Ronald Maddox, Dean of the Campbell University School of Pharmacy. “The FDA determined that these two drugs are not therapeutically equivalent and, therefore, has not listed the products with a therapeutic equivalence code.”‘
**Defending Cardiovascular Benefits**	
Mosca L. Hormone Replacement Therapy in the Prevention and Treatment of Atherosclerosis.Curr Atherosclerosis Reports 2000 Jul;2(4):297-302.	“Remarkable consistency among epidemiologic studies supports a cardioprotective role of ERT.”“The biologic evidence for a role of estrogen to prevent CVD is compelling. Concerns regarding potential adverse effects among susceptible women and the lack of confirmatory data from randomized trials make general recommendation [sic] difficult to make.”
Rackley CE. New clinical markers predictive of cardiovascular disease: the role of inflammatory mediators. Cardiol Rev. 2004;12(3):151-7.	“The use of HT was associated with higher baseline levels of CRP but no change in IL-6 in either the case or the control group. However, the use of HT was less important than the actual baseline values of CRP and IL-6 in predicting cardiovascular risk.”
Koh KK. Can a Healthy Endothelium Influence the Cardiovascular Effects of Hormone Replacement Therapy? Int J Cardiol. 2003;87(1):1-8.	“… the HERS trial had certain methodological pitfalls, including insufficient statistical power, a high crossover rate between treatment arms, and other medications effect. Second, the early increment in coronary event rates might have been precipitated by procoagulant effects of HRT and a susceptible cohort… The controversy occasioned by the HERS trial can be resolved only through sufficiently powered, randomized controlled trials.”
**Positioning Low-dose Therapy**	
Lobo R, Whitehead M. Is Low-Dose Hormone Replacement Therapy for Postmenopausal Women Efficacious and Desirable?Climacteric. 2001 Jun;4(2):110-9.	“The potential for fewer side-effects with low-dose formulations may play an important role in enhancing patient acceptance and continuance of ERT/HRT. Lower doses may also reduce patients’ concerns about cancer.”
Maddox RW. The Efficacy and Safety of Low-dose Hormone Therapy. US Pharmacist 2004 (June).	“The recent approval by the FDA of the new oral LD [low-dose]-ET/HT formulations… represents an important advance in menopausal management and osteoporosis prevention. The dosage of ethinyl estradiol in low-dose oral contraceptives is… four to seven times greater than that in SD [standard-dose]-HT, or six to 14 times greater than that in LD-ET/HT.”

Article	Planned “Potential Key Messages” [20]	Messages From Published Article
Utian WH, Shoupe D, Bachmann G, Pinkerton JV, Pickar JH. aRelief of vasomotorsymptoms and vaginal atrophy with lower doses of conjugated equine estrogens andmedroxyprogesterone acetate. Fertil Steril. 2001 Jun;75(6):1065-79.	”This paper will serve as the lead paper and contain the entire list of HOPE trial investigators. The suggestions contained in this paper for treatment strategies will be reiterated in each of the four publications.””When compared to Prempro TM 2.5, the 0.45/1.5 HRT dose provides similar symptom relief and results in a similar vaginal maturation index. This product will be suggested as the preferred dose for patients starting HRT.””The 0.3/1.5 HRT dose also provides symptom relief, although it may be somewhat less than with the 0.45/1.5 dose and Prempro 2.5. The vaginal maturation index is similar to the 0.45/1.5 dose and Prempro 2.5. This product will be suggested for older (>65 years), new-start patients and those who may require a lower dose (eg, because of side effects, fear of breast cancer).”	“In summary, the Women’s Hope trial demonstrated the efficacy of CEE 0.45 combined with either 1.5 or 2.5 mg of MPA per day and of CEE 0.3/MPA 1.5 for vasomotor symptom relief and improvement in VMI in postmenopausal women. Confirmation of the efficacy of these lower doses of estrogen and progestin expands the number of options for women considering HRT. Lower doses of CEE and CEE/MPA appear to be as effective as the most commonly prescribed doses and should be considered as initial treatment options for a majority of women. These lower-dose combinations may allow more patients to obtain the proven preventive benefits of long-term HRT.”Note: Acknowledges the “editorial assistance provided by Bernadette Janas, Ph.D.”
Pickar JH, Yeh I-T, Wheeler JE, Cunnane MF, Speroff L. bEndometrial effects of lower doses of conjugated equine estrogens and medroxyprogesterone acetate: two-year substudy resultsFertility and Sterility 2003;80(5):1234-1240	”Both the 0.45/1.5 and 0.3/1.5 doses demonstrate similar efficacy with respect to endometrial safety. A hyperplasia rate of 0.37% (1 case) was reported for both groups. For women in the Prempro 2.5 group, no cases of endometrial hyperplasia were observed.”	“The incidence of endometrial hyperplasia was significantly lower (P≤.001) for the groups treated with CEE/MPA than with the comparable dose of CEE alone, with the exception of the lowest dose (CEE 0.3/MPA 1.5 mg), which did not reach statistical significance in the consensus analysis.”“Moreover, since low-dose regimens of E, appropriately balanced with progestin, are able to provide benefit while maintaining endometrial protection, the combinations CEE 0.45/MPA 1.5 mg and CEE 0.3/MPA 1.5 mg may be preferable to commonly prescribed HT combinations.”Note: acknowledges “editorial assistance of Karen Mittelman, PhD”
Archer DF, Dorin M, Lewis V Schneider DL, Pickar JH. cEffects of lower doses of conjugated equine estrogens and medroxyprogesterone acetate on endometrial bleeding.Fertility and Sterility 2001;75(6):1080-1087.	”When compared to Prempro 2.5, the 0.45/1.5 HRT dose results in an improved rate of amenorrhea, particularly in the early cycles.””The rates of amenorrhea for women receiving the 0.3/1.5 HRT dose were higher than those observed for women receiving the 0.45/1.5 HRT dose, although these differences were not statistically significant (based on initial data presented).”	“The increased rates of amenorrhea that we observed in women treated with lower doses of CEE and MPA compared with those taking the most commonly prescribed CEE/MPA regimen provide strong evidence that lower-dose HRT reduces vaginal bleeding.”“In conclusion, lower doses of CEE and MPA produced higher rates of amenorrhea than the most commonly prescribed doses, especially during earlier cycles of therapy. Our results suggest that these lower-dose regimens are an appropriate choice of therapy in newly menopausal patients. ““Furthermore, because the effects of these regimens on bleeding profiles became more pronounced with time, our study provides valuable information that may help more women achieve the benefits of HRT over a longer period of time. “Note: acknowledges “Bernadette Janas, Ph.D., for editorial assistance.”
Lobo RA, Bush T, Carr BR, Pickar JH.^d^Fertility and Sterility, July 2001; 76(1):13-23.Effects of lower doses of conjugated equine estrogens and medroxyprogesterone acetate on plasma lipids and lipoproteins, coagulation factors, and carbohydrate metabolism	”When compared to Prempro 2.5, the 0.45/1.5 HRT dose results in similar increases in HDL-cholesterol and similar decreases in LDL-cholesterol. Carbohydrate metabolism was not adversely affected by either treatment.”	“In summary, this study indicates that lower doses of CEE and CEE/MPA induce favorable changes in lipoproteins and modest changes in carbohydrate metabolism and hemostatic factors.”“A regimen of 0.45 mg of CEE alone or in combination with 1.5 mg of MPA offers a lower dose of HRT and a metabolic profile similar to the HRT dose most commonly prescribed today. Although we anticipate that the metabolic benefit observed in this younger postmenopausal population would pertain to older women as well, this supposition will require confirmation in future studies.”Note: acknowledges “editorial assistance of Karen Mittleman, Ph.D…”

*For documentation of ghostwriting, see Table S2.

**Box 1. Ghostwritten Reviews and Commentaries on Hormone Therapy**

DesignWrite helped Wyeth create ghostwritten reviews and commentaries to:

- Mitigate perceived risks of hormone-associated breast cancer
- Promote unproven, off-label uses, including prevention of dementia, Parkinson's disease, and visual impairment
- Raise questions about the safety and efficacy of competing therapies (competitive messaging)
- Defend cardiovascular benefits, despite lack of benefit in RCTs
- Position low-dose hormone therapy

Table 1 provides details of these articles and their key messages.

In addition, clinical trial reports were sometimes modified for marketing purposes. For example, Wyeth apparently wanted the metabolic effects of a Premarin/trimegestone combination removed from the lead publication on this product. A 2003 DesignWrite email to James H. Pickar, a physician employed by Wyeth, noted the marketing team's concerns: “… it is highly desirable for them to not have the metabolic data included in the lead paper, as this would cause labeling problems, making the lead paper unusable for promotional purposes” [33].

## Managing “Authors” and Journals

An important part of DesignWrite's work for Wyeth was to manage “authors” and journals. There is evidence in unsealed DesignWrite documents that although some authors signed off on ghostwritten articles, others insisted on contributing to their articles. One co-author seemed puzzled by the concept that she was to author, but not write, an article [34]: “From what you have written, I would be more of an ‘editor’ rather than the major writer—that is, you guys would be writing the versions—with me ‘altering, editing, etc.? Is that correct?’” This query was in response to an e-mail from Karen Mittleman (a DesignWrite employee who supervised medical writers) that stated: “The beauty of this process is that we become your postdocs! … We provide you with an outline that you review and suggest changes to. We then develop a draft from the final outline. You have complete editorial control of the paper, but we provide you with the materials to review/critique” [34].

After receiving a draft, this co-author (Leiblum) noted that the outline contained “…many factual errors and mis-information (sic), as well as over-emphasis on the hormonal contributions to post-menopausal sexuality as opposed to the interpersonal contributions” [35]. She did not agree to authorship until her numerous changes [36] were incorporated [37]. To appease another author, a writer was told by DesignWrite that the author's “…own additions will probably have to stay no matter what” [38]. This author later unsuccessfully attempted to credit the ghostwriter as a coauthor[39].

In general, authors' revisions were permitted if marketing messages were not compromised. For example, at a 2002 Strategic Publications Development Meeting, an author's request “to shorten the Early Bone Loss paper…and prepare it for a practical audience…” was discussed [40]. The consensus was that this was acceptable as long as the message remained that “HRT is the most cost-effective therapy for preventing bone loss for women entering menopause due to its other benefits and low cost” [40].

Furthermore, when one author submitted a manuscript “unilaterally” to a journal, an attempt was made by DesignWrite to reassert control: “We have provided him with an updated draft of the manuscript and he will try to incorporate these revisions in the paper where possible…” [41].

The trivial role authors were expected to play is demonstrated by DesignWrite's reference to planned reviews as “opinion leader–endorsed” [42]. Furthermore, authors were considered interchangeable; one document states, “I moved Dr. Creasman as an author to the patient ed piece (with Blackwood, Weiss, & Speroff) and left Horwitz and Boman on the basic science manuscript” [43], although Horwitz's name does not appear on the published article.

Finally, in response to a question about whether previously commissioned papers could be reused, Gerald Burr of Wyeth wrote: “You can't just put another name on the article, but you can plagiarize the way we did when we wrote papers in college. What you need to do is give your potential authors Karen's version of the article before the author modified it. Then have your authors modify it for publication under their name. Wyeth owns Karen's draft, not the final publication” [44]. Burr supplied five drafts [45] but asked that Karen Mittleman be notified of the plans for reuse “so she can advise if we are going to piss off any of the U.S. authors” [44].

DesignWrite's ghostwriters also managed journals by responding to editor and reviewer comments [46],[47]. Ghostwriters argued for retention of specific marketing messages, sometimes scolding reviewers under the guise of defending peer-review. Responses to one presumably unfavorable review included: “The review of the current paper is not the appropriate place to criticize the methodologic flaws of published papers”; and “The reviewer's suggestion to revise the statement on page 8 ‘…absence of a definitive causal relationship between exogenous postmenopausal ERT [estrogen replacement therapy] and breast cancer risk’ is not justified. This interpretation is well documented” [46].

In one case, a ghostwriter asked the author for assistance in preparing a response: “…If you have any thoughts about how we might reply to this reviewer's comment, please let us know.” The author provided a slide to the writer: “the enclosed powerpoint could serve as a figure to summarize how this all hangs together… it obviously needs ‘cleaning up.’” [48].

## Messaging

Clinical trials, reviews, case reports, letters, and other publications are used by pharmaceutical companies to convey specific marketing messages. Besides extolling the benefits of a specific drug, marketing messages may emphasize the prevalence or severity of targeted conditions, promote unproven uses, deride competing therapies, or reassure clinicians that adverse effects are rare, manageable, or not specific to a targeted therapy.

Even though a 1997 DesignWrite proposal admitted that “HRT continues to be a drug in search of a disease” [49], my examination of the available documents indicates that the lack of evidence regarding the prevention and treatment of cardiovascular disease, dementia, and other diseases proved no deterrent to Wyeth/DesignWrite's promulgation of numerous marketing messages positioning HT as a panacea. A message strategy listed under “Value of Estrogen Therapy (or Bundle of Benefits)” in DesignWrite's 1997 publication plan was “Define the serious nature of menopause-related illness and demonstrate the *clinical* benefits of instituting hormone replacement therapy in the treatment of multiple disorders including cardiovascular, osteoporosis, vasomotor, Alzheimer's, and colon cancer” [15].

### Defending Cardiovascular Benefits

Soon after HERS found no evidence for cardiovascular benefit for HT, numerous articles attacking the trial appeared in the medical literature. A 2001 article authored by Thorneycroft [50] states: “The results of HERS do not contradict the weight of epidemiologic study findings showing a primary protective CVD effect in longer-term HRT users. Indeed, because of possible serious flaws in the study, a protective benefit of HRT for secondary CVD prevention cannot be ruled out.” Some articles were ghostwritten (see Table S1). For example, a 2000 article authored by Mosca [51] states, “Remarkable consistency among epidemiologic studies supports a cardioprotective role of ERT.”

### Saving One's Skin and Self-Esteem

After the WHI lay to rest the concept that HT prevented cardiovascular disease, stroke, and Alzheimer's, marketing messages shifted to unproven lifestyle benefits (see Table 1). Messages in the 2003 publication plan included: “the importance of quality-of-life issues that are improved with postmenopausal HT use” and “…the benefits of postmenopausal HT on skin and sexual health” [52]. Ghostwritten articles supporting this message included a 2005 article by Brincat [53] that states, “Estrogen treatment in postmenopausal women has been repeatedly shown to increase collagen content, dermal thickness, and elasticity.” A 2004 article by Bachman and Leiblum states, “Continual estrogen loss often leads to numerous signs and symptoms, including changes in the vascular and urogenital systems. Alterations in mood, sleep, and cognitive functioning are common as well. These changes may contribute to lower self-esteem, poorer self-image, and diminished sexual responsiveness and sexual desire” [54].

### Questioning Breast Cancer Risk

Many ghostwritten articles dispute the link between HT and breast cancer, or imply, falsely, that breast cancers associated with HT are less aggressive (see Tables 1 and 2, and Box 2). Some articles were built around a single message, including a 2003 paper by Eden [55]. Notes from a publication planning meeting held in 2000 read: “…John Eden was suggested as the author of a breast cancer paper questioning the role of progestins as a causative factor” [56]. Discussion points the ghostwriter was told to put in the paper included “why progestins may not be responsible for the incidence of breast cancer in hormone replacement therapy (HRT) users” [57]. The published article states, “…results from epidemiologic studies are inconsistent and mechanistic studies have not provided a physiologic foundation to implicate progestin in the pathogenesis of breast cancer” [55].

**Table 2:** Relationship between planned messages and final text in the supplement *Postmenopausal Hormone Therapy and Breast Health: A Review for Clinicians*.

Article	Planned Messages [80]	Excerpts From Published Article [109]
Speroff L.^a^Inconsistency in Epidemiologic Findings on Postmenopausal Hormone Therapy and Breast Cancer	“Recent studies suggest the possibility of a slightly increased risk of breast cancer associated with long-term use of postmenopausal hormone therapy”“However, the results from the many epidemiologic studies on this relationship are not consistent or uniform, and taken together, fail to provide definitive evidence of causality”“Discussion of results of recent studies, pointing out strengths and weaknesses, as well as both null and positive findings of a relationship between HRT and breast cancer detection”“Mortality data, detection bias, more treatable tumors”	“…more than half of the studies conducted in the past 25 years found either no difference in risk or a decreased risk of breast cancer with ERT/HRT use.”“…if there is an increased risk of breast cancer associated with the use of ERT/HRT, this risk must be small.”“These recent studies continue the pattern of inconsistency in research on this topic…”“Even studies that detect an increased risk of breast cancer in hormone users suggest, paradoxically, a better outcome.”“In the absence of results from large, randomized clinical trials, clinicians can help patients to understand that current research findings on breast cancer risk and long-term use of ERT/HRT are inconclusive, no studies find an increased risk with short-term use, and women who use postmenopausal hormones have lower mortality rates.”
DiSaia PJ.^b^A Rationale for Estrogen Use in Breast Cancer Survivors [originally Estrogen use after breast cancer]	“Rationale for estrogen use in breast cancer survivors (esp., number of women)”“Review evidence from naturally-occurring situations with estrogen exposure and breast cancer (pregnancy during or after breast cancer, HRT use, OC use)”“Use of estrogen by women who have had breast cancer does not appear to increase risk of recurrence”	“Numerous studies have reported better survival rates for women using HRT at the time of breast cancer diagnosis compared with those for nonusers.”“Observational studies suggest that postmenopausal hormone therapy after breast cancer diagnosis does not negatively affect breast cancer recurrence or survival.”“Breast cancer prognosis is not negatively affected by exposure to increased estrogen levels during or after pregnancy or by exposure to exogenous estrogens around the time of diagnosis.”“… exposure to estrogen around the time of breast cancer diagnosis and the use of ERT/HRT in breast cancer survivors do not negatively impact patient outcomes.”
Commonly Asked Questions About Postmenopausal Hormone Therapy and Breast Health [Originally Patient Education Handout]^c^	“Inform patients that many studies do not show an increased risk”“give clear information about how many more women will get breast cancer if reported risk are [sic] accurate”“compare risk of breast cancer from postmenopausal hormone therapy with everyday risks”“emphasize significant health risks for postmenopausal women (cardiovascular diseases)”“Connect HRT to OCs and the comfort level that many women have with OCs”	“Close to 60 research studies have compared breast cancer risk in women who use HRT and in women who do not. Most of these studies found no increased risk of breast cancer with HRT use.”“Researchers have consistently found no increase in breast cancer risk with short-term use of HRT. Studies on long-term use, however, have reported conflicting results, which means that more studies are needed.”“Researchers have consistently found that HRT use does not increase breast cancer risk in women with a family history of breast cancer.”“However, there is no evidence that HRT use affects breast cancer detection.”“Studies have found that breast cancer patients using HRT at the time they were diagnosed tend to have smaller tumors that are less aggressive and are detected at a more favorable stage than are tumors of nonusers.”“…estrogen and progesterone, are the same hormones found in birth control pills, only at much lower doses (less than 1/10th the dose).”“Use of HRT also protects bone health and may decrease a woman's risk of developing colon cancer, Alzheimer's disease, heart disease, and macular degeneration (a condition associated with aging that may cause loss of vision).”

All documentation of ghostwriting is taken from Szaller J. Wyeth's hormone therapies & ghostwritten medical literature (unpublished manuscript), with permission.

a DWRITE078512; DWRITE078370; Janas_010408 at 483:11–485:13.

b Draft outline DWRITE078245; Janas_010408 at 471:13–472:3 and 477:21–479:22.

c DWRITE001221 and DWRITE078847 at DWRITE078850; Janas_010408 at 432:5–9 and 460:10–461:5.

**Box 2. Planned Messages and the Final Text in the Supplement *Postmenopausal Hormone Therapy and Breast Health: A Review for Clinicians***

Articles in this supplement, which also included a patient education handout,

- Cast doubt on the link between HT and breast cancer
- Questioned whether HT-induced changes in mammographic density were related to increased breast cancer risk
- Implied that use of estrogen after breast cancer was safe
- Promoted the concept that HT-associated breast cancers were less aggressive cancers

Table 2 details how the numerous planned messages included in the Outline for this supplement [109] were incorporated into the published articles [80] by providing relevant quotations from both sources.

### Battling Competitors

Ghostwritten articles also raise questions about the safety of competing drugs and the efficacy of generics (see Table 1). For example, negative messages were developed for raloxifene, a selective estrogen receptor modulator (SERM) used to treat osteoporosis [58]. Raloxifene was to be cast as a drug that could worsen hot flashes and for which long-term effects were not known. The specter of tamoxifen, an earlier SERM that increased uterine cancer risk, would be raised [58]. A 1999 ghostwritten article by Curtis states: “…the risk:benefit of the emerging SERMs needs to be better defined and evaluated. In light of the suggestion that many menopausal women seek medical attention because of vasomotor symptoms, the potential exacerbation of the symptoms with SERMs would not be advantageous in this patient group” [59]. However, because Wyeth was developing its own SERM, it was subsequently decided that DesignWrite would suggest that “future SERMs may be better” [60]. In line with this decision, a 2001 article on SERMs by Curtis states: “The development of future generations of SERMS that improve upon the current therapies is eagerly anticipated” [61].

Negative messages were also developed for alternative therapies and generic drugs. For example, an article was planned that would “stress the fact that alternative therapies have increased in usage since the WHI even though there is little evidence that they are effective or safe…” [52], and a 2001 article by Ansbacher states, “Generic conjugated estrogens have been manufactured; however, the therapeutic equivalence of these generic products to CEE cannot be ensured…” [62] (see Table 1).

Finally, although the unique benefits of Premarin products were emphasized, any risks associated with them were cast as applying to all HT products. A 2003 publication program document suggested highlighting “the class effects of all HT products” [52]. Subsequently, a 2004 article by Warren states, “Overall, these data indicate that the benefit/risk analysis that was reported in the Women's Health Initiative can be generalized to all postmenopausal hormone replacement therapy products” [63].

Table 1 lists other examples of marketing messages included in ghostwritten reviews. In addition, Tables 3 and S1 summarize planned and published marketing messages in ghostwritten articles for clinical trials of low-dose Prempro and of Premarin with trimegestone, respectively. Wyeth ceased development of this latter combination in 2003 [64]. It is important to note that the Tables provided as supporting evidence for this Policy Forum article only list articles for which extensive documentation of ghostwriting exists within publicly available documents. These articles and their authors may represent only the tip of the iceberg.

**Table 3:**
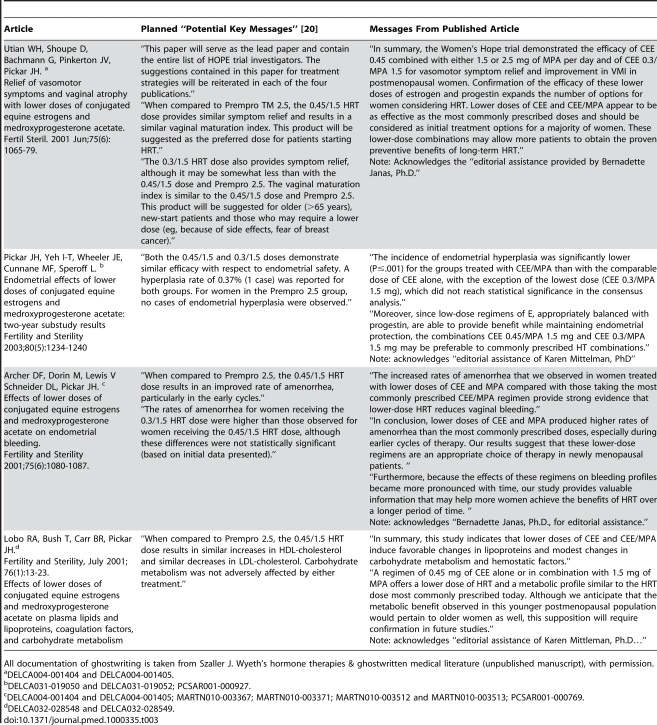
Planned and published messages in the ghostwritten HOPE trials of low-dose Prempro.

All documentation of ghostwriting is taken from Szaller J. Wyeth’s hormone therapies & ghostwritten medical literature (unpublished manuscript), with permission.

a DELCA004-001404 and DELCA004-001405.

b DELCA031-019050 and DELCA031-019052; PCSAR001-000927.

c DELCA004-001404 and DELCA004-001405; MARTN010-003367; MARTN010-003371; MARTN010-003512 and MARTN010-003513; PCSAR001-000769.

d DELCA032-028548 and DELCA032-028549.

## Supplements

Another way that pharmaceutical companies spread their marketing messages is through supplements—separately bound publications carrying a medical journal's name that are often industry-sponsored and rarely peer-reviewed. In DesignWrite's words: “The value of journal supplements is that it allows you to better tailor your marketing message since it is a manufacturer-sponsored publication form. Additionally, reprints of supplements may be purchased and distributed widely among health care professionals via sales representatives…” [65].

Perhaps because meeting proceedings lend credibility to supplements, Wyeth/DesignWrite held an “Expert Forum on Breast Cancer Health” in April 2001 in Philadelphia [66] to develop materials for a CME supplement [67]. Wyeth/DesignWrite invited speakers [68], assigned topics [67],[69], provided participants with a “reading packet” [70], and an agenda [71],[72] that listed the topics the speakers should address. These topics seemed designed to reassure clinicians that breast cancer risk with HT was extremely low and that breast cancers in women on HT were easily treated. The “key messages to be derived from those talks” [69],[71] aimed to “diminish the negative perceptions” [15] regarding HT and included: “The evidence that use of ERT and/or HRT increases risk for breast cancer is weak”, “MPA does not increase risk of breast cancer”, and “Women who have had breast cancer may gain benefits from ERT/HRT” [71]. DesignWrite prepared drafts of the supplement articles based on the speaker' slide presentations [73],[74] and submitted them to the journal *Women's Health in Primary Care* [75]. DesignWrite responded to comments from the University of Wisconsin [76] (the CME accreditor) and two reviewers from the journal [76]–[78], one of whom subsequently authored a ghostwritten article for Wyeth/DesignWrite [79] (see Table 1). DesignWrite also asked Jeff Solomon of Wyeth's marketing department to provide “comments or suggestions” to reviewers' comments [76].

### Better Breast Cancers

The resulting supplement, *Postmenopausal Hormone Therapy and Breast Health: A Review for Clinicians* [80], included unsupported claims that HT decreased mortality and had multiple health benefits, but its predominant marketing message appears to be the mitigation of concerns that HT causes breast cancer (Box 2 and Table 2). Speroff declares in one article, “…if there is an increased risk of breast cancer associated with the use of ERT/HRT, this risk must be small”. Fiorica states in another article, “...there is no evidence that ERT/HRT-induced changes in breast density, which are rapidly reversible upon cessation of hormone therapy, increase breast cancer risk”, states Fiorica in another article. A breast cancer diagnosis was, apparently, no reason to cease use. DiSaia states, “Observational studies suggest that postmenopausal hormone therapy after breast cancer diagnosis does not negatively affect breast cancer recurrence or survival.” Similarly, Fiorica states: “Women who use ERT/HRT after breast cancer diagnosis may also have more favorable outcomes compared with nonusers” [80].

Commenting on drafts of the supplement's introduction, Jamie Durocher of Wyeth Marketing [81],[82] suggested: “So that physicians are open to reading the supplement, I think certain revisions are necessary to unobtrusively acknowledge the conflict of recent years (without being negative)” [80]–[83]. Regarding the patient handout, Durocher noted: “…(any risk of cancer is perceived as too much) it may be helpful to also mention in the first answer that women on HRT who do develop cancer have a less virulent cancer and a better outlook for recovery…” [84].

### Promotion via Exam

The CME test accompanying the supplement reinforced its marketing messages. For example, based on the text, the answer to the test question, “One of the most consistent findings from research on postmenopausal hormone therapy and breast cancer risk is that:” is most likely to be “ERT/HRT use is associated with a decrease in all-cause mortality”. The most likely answer to the question, “Use of ERT/HRT has traditionally been avoided in breast cancer survivors because of:” is “the unsubstantiated hypothesis that hormone therapy will activate dormant malignant cells” [80]. The CME accreditor claims that it has no records of the correct answers to this 2002 test [85].

Wyeth paid $413,140.60 for the meeting, supplement, and CME accreditation [86]. The supplement was mailed to 128,000 physicians [87] with regular and “Gynecology Editions” of *Women's Health in Primary Care*. Wyeth bought 1,500 additional copies for distribution to its sales force [86] and distributed the supplement to media and “select thought leaders” [88].

The supplement acknowledges support “…by an unrestricted educational grant from Wyeth-Ayerst Pharmaceuticals” [80] and includes the disclaimer: “The opinions expressed in the articles that appear in this supplement are those of the authors, and do not necessarily reflect those of *Women's Health in Primary Care* or Wyeth-Ayerst Laboratories” [80]. DesignWrite, which received $25,000 per article [89], is not mentioned.

## Discussion

Marketing messages in credible journals have almost certainly contributed to widespread use of HT among millions of women who had no medical indication for the drug. Journal articles were mailed or delivered via drug reps to doctors. DesignWrite documents also indicate that the supplement and at least seven other ghostwritten publications were to be distributed to Medical Science Liaisons—physicians or pharmacologists employed by Wyeth to respond to physician queries [90]–[94].

Ghostwriting has been documented for drugs other than Prempro. For example, Forest Laboratories' 2004 marketing plan for Lexapro (escitalopram) [95], stated: “Bylined articles will allow us to fold Lexapro messages into articles on depression, anxiety and comorbidity developed by (or ghostwritten for) thought leaders” [96]. Ghostwriting has also been documented in the promotion of Paxil (paroxetine) [97]–[100], “Fen-phen” (fenfluramine and phentermine) [101], Neurontin (gabapentin) [102], Vioxx (rofecoxib) [103], and Zoloft (sertraline) [104].

Industry-funded marketing messages may infest articles in every medical journal. Although the prevalence of proffered or accepted invitations to sign ghostwritten articles is unknown, the practice may be common. Several recent examples of academic physicians receiving invitations to affix their names to prewritten articles have been documented [11],[105]–[106]. Acceptance of ghostwriting, euphemistically termed “editorial assistance,” may be so widespread that it is considered normal. This could explain why several authors of ghostwritten articles have defended their involvement [107],[108].

Medicine, as a profession, must take responsibility for this situation. Naïveté is no longer an excuse. Perhaps physician-investigators should create and uphold a standard where relationships with industry are regarded as unsavory rather than sought after. Academic institutions and medical journals should take a hard line on ghostwriting. Patient care will benefit if physicians draw together as a profession to denormalize relationships with industry and avoid the role of corporate pawns in the future.

## Supporting Information

Table S1 Planned marketing messages consistent with published text in clinical trials of estrogen and trimegestone.
(0.08 MB DOC)

Table S2 Examples of ghostwritten reviews and commentaries.
(0.07 MB DOC)

## Abbreviations

CEE: conjugated equine estrogens
CVD: cardiovascular disease
ERT: estrogen replacement therapy
FDA: U.S. Food and Drug Administration
HERS: Heart and Estrogen/progestin Replacement Study
HT: menopausal hormone therapy
HRT: hormone replacement therapy
MECC: medical education and communication company
MPA: medroxyprogesterone acetate
RCT: randomized controlled trial
SERM: selective estrogen receptor modulator
WHI: Women's Health Initiative
