# Supplementary material for: The Haunting of Medical Journals: How Ghostwriting Sold “HRT”
Source: PLoS Med. 2010 Sep 7;7(9):e1000335. doi: 10.1371/journal.pmed.1000335 (PMC2935455; doi:10.1371/journal.pmed.1000335)
Supplement: Table S1 — Planned marketing messages consistent with published text in clinical trials of estrogen and trimegestone. (0.08 MB DOC) [file pmed.1000335.s001.doc]

**Table S1. Planned marketing messages consistent with published text in clinical trials of estrogen and trimegestone * [1] .**

| **Message #4**  “Age and estrogen deficiency in menopausal women results in: vasomotor symptoms, genitourinary symptoms, increased risk for osteoporosis, increased risk for cardiovascular disease, cognitive decline, overall decrease in quality of life.” | |
| --- | --- |
| **Article** | **Excerpts** |
| Pornel B, Spielmann D.**^a^**  A study of the control of climacteric symptoms in postmenopausal women following sequential regimens of 1 mg 17beta-estradiol and trimegestone compared with a regimen containing 1 mg estradiol valerate and norethisterone over a two-year period. Gynecol Endocrinol. 2005 Aug;21(2):74-81. | “The effects of estrogen deficiency in postmenopausal women manifest themselves clinically as a wide spectrum of symptoms of neuroendocrine origin.”  “Besides experiencing hot flushes and night sweats, most menopausal women also suffer from other psychological and/or somatic symptoms such as insomnia, nervousness, headaches, anxiety, vaginal dryness and depression. These symptoms affect a large number of women, lowering their quality of life…” |
| Koninckx PR, Spielmann D.**^b^**  A comparative 2-year study of the effects of sequential regimens of 1 mg 17beta-estradiol and trimegestone with a regimen containing estradiol valerate and norethisterone on the bleeding profile and endometrial safety in postmenopausal women. Gynecol Endocrinol. 2005 Aug;21(2):82-9. | “The most effective treatment for menopausal symptoms, which are caused by depletion in estrogen levels, is hormone replacement therapy (HRT). The  main goal of HRT is the relief of vasomotor  symptoms, although there is evidence for other benefits, such as protection against bone loss and alleviating other typical menopausal symptoms (e.g., sleep and psychological disturbances).” |
| Bouchard P, De Cicco-Nardone F, Spielmann D, Garcea N and the Trimegestone 301 Study Group.**^c^**  Bleeding profile and endometrial safety of continuous combined regimens 1 mg 17beta-estradiol/trimegestone versus 1 or 2 mg 17beta-estradiol/norethisterone acetate in postmenopausal women. Gynecol Endocrinol. 2005 Sep;21(3):142-8. | “The decline in endogenous estrogen level at the time of menopause is associated with a wide range of symptoms, including hot flushes, night sweats, mood changes and sleep disturbances.” |
| Gambacciani M, Spielmann D, Genazzani AR.**^d^**  Efficacy on climacteric symptoms of a continuous combined regimen of 1 mg 17beta-estradiol and trimegestone versus two regimens combining 1 or 2 mg 17beta-estradiol and norethisterone acetate. Gynecol Endocrinol. 2005 Aug;21(2):65-73. | “Menopause is an estrogen-deficient state that can lead to profound physiological and psychological changes in women. Early-onset symptoms associated with menopause include hot flushes, night sweats and insomnia, as well as psychological disturbances such as depression and anxiety.” |
| Meuwissen JHJM, L. Beijers-De Bie T, Vihtamaki R, Tuimala N, Siseles C, Magaril HS, Houben PWH, Murga C, Spielmann D, De Villiers TJ.**^e^**  Assessment of the metabolic tolerance in postmenopausal women over a 1-year period of two hormone replacement therapies containing estradiol in combination with either norgestrel or trimegestone, Gynecol Endocrinol 2002; 16:155-162. | “The loss of ovarian function at menopause and the subsequent depletion of blood and tissue estrogen levels have significant detrimental effects on risk factors for cardiovascular disease.” |
| **Message #10**  “New HRT regimens with improved progestins to provide alternate management options while based on the clinical experience of established estrogens are needed.” | |
| **Article** | **Message As Published** |
| Pornel B, Spielmann D. | “However, the addition of a progestin can alter the clinical profile of a hormone replacement therapy (HRT) preparation; therefore, preparations with different progestins are useful in order to allow the most well tolerated preparation to be chosen for an individual woman.” |
| Koninckx PR, Spielmann D. | N/A |
| Bouchard P, De Cicco-Nardone F, Spielmann D, Garcea N and the Trimegestone 301 Study Group. | “The 1 mg 17ß-E_2_/0.125 mg TMG continuous combined regimen provides a new therapeutic option for postmenopausal women considering HRT for menopausal symptom relief.” |
| Gambacciani M, Spielmann D, Genazzani AR. | “A significant clinical benefit could be achieved by developing a highly selective progestin with strong endometrial protection, a good bleeding profile, and little effect on other body systems.” |
| Meuwissen JHJM, L. Beijers-De Bie T, Vihtamaki R, Tuimala N, Siseles C, Magaril HS, Houben PWH, Murga C, Spielmann D, De Villiers TJ. | “The difference in the lipid profiles between the treatment groups in this study can be explained by the properties of the two progestogens used; norgestrel has an androgenic effect, which prevents the estrogen-induced increase in HDL cholesterol and even induces a decrease in HDL cholesterol, and trimegestone, which exhibits no androgenic effect.”  “The results of the present study suggest that the use of trimegestone may be preferable to norgestrel in combination with estradiol, because of the improved lipid profile.” |
| **Message #12**  “The preclinical profile of trimegestone indicates that it has progesterone receptor selective binding and blocks the unwanted actions of estrogens in the uterus without blocking the beneficial actions of estrogens on bone and hot flush [sic]”. | |
| **Article** | **Message As Published** |
| Pornel B, Spielmann D. | “Trimegestone (TMG) is a novel 19-norpregnane progestin. It shows a high affinity and potency for the  progesterone receptor and high selectivity for the endometrium…” |
| Koninckx PR, Spielmann D. | “Trimegestone (TMG) is a novel 19-norpregnane derivative with a high relative binding affinity for the progesterone receptor and high selectivity for the endometrium. At clinically relevant doses, it is strongly progestogenic but also very specific…”  ”Thus, a hormone therapy that combines 17ß-estradiol (17ß-E_2_) with TMG should prevent endometrial hyperplasia and cause minimal interference with the beneficial effects of estradiol.” |
| Bouchard P, De Cicco-Nardone F, Spielmann D, Garcea N and the Trimegestone 301 Study Group. | “Trimegestone (TMG) is a novel 19-norpregnane derivative with a high relative binding affinity for the progesterone receptor and selectivity to the endometrium.” |
| Gambacciani M, Spielmann D, Genazzani AR. | “*In vivo*, TMG has shown strong progestogenic activity in rodents, especially in the endometrium…”  “Together, the data indicate that TMG is a very potent and selective progestin, with an overall improved pharmacological profile compared with other available progestins.” |
| Meuwissen JHJM, L. Beijers-De Bie T, Vihtamaki R, Tuimala N, Siseles C, Magaril HS, Houben PWH, Murga C, Spielmann D, De Villiers TJ. | “A new HRT preparation combines estradiol with trimegestone. The latter compound, 17ß[(S)-2-hydroxypropanoyl]-1 7-methyl estra-4,9 dien 3 one, is a novel norpregnane progestogen that exhibits a favorable pharmacological profile. It shows a high affinity for the progesterone receptor and a potent progestomimetic activity, possesses no androgenic, glucocorticoid or mineralocorticoid activity, and neither has an affinity for the estrogen receptor nor exhibits uterotrophic activity.” |
| **Message #16**  “The combination of Premarin/trimegestone* is an effective HRT regimen that affords adequate endometrial protection and relief of the vasomotor symptoms of menopause with a bleeding profile comparable or slightly better than other HRT regimens.” | |
| **Article** | **Message As Published** |
| Pornel B, Spielmann D. | “In addition to its efficacy in relieving climacteric symptoms, this regimen has also been shown to provide adequate protection on the endometrium based on the recommendations of the European Agency for the Evaluation of Medicinal Products, have an overall favorable bleeding profile, and be well tolerated.” |
| Koninckx PR, Spielmann D. | “The results of this study indicate that the 1 mg 17ß-E_2_/0.25 mg TMG sequential regimen provides adequate protection on the endometrium according to EMEA recommendations, with an overall more favorable bleeding profile and a significantly lower number of bleeding days than the 1 mg 17ß-E_2_/0.125 mg TMG regimen. It is also well tolerated and generally as safe as the 1 mg E_2_V/1 mg NET comparator.” |
| Bouchard P, De Cicco-Nardone F, Spielmann D, Garcea N and the Trimegestone 301 Study Group. | “…the continuous combined regimen of 1 mg 17ß-E_2_ and 0.125 mg TMG provides endometrial protection, as no cases of hyperplasia were detected throughout the  2 years of the study. This protective effect was similar to that exerted by the 17ß-E_2_/NETA comparators,  and comparable to that reported by previous studies for 0.625 mg conjugated equine estrogens (CEE)  and 5–10 mg medroxyprogesterone acetate (MPA).  Furthermore, it is also consistent with the findings from the Women’s HOPE (Health, Osteoporosis, Progestin, Estrogen) study…” |
| Gambacciani M, Spielmann D, Genazzani AR. | “In conclusion, this study demonstrates that 1 mg 17ß-E_2_/0.125 mg TMG has a rapid onset of action in effectively improving menopausal symptoms.”  “In the accompanying paper, the 1 mg 17ß-E2/0.125 mg TMG regimen is also shown to provide adequate protection of the endometrium based on the European Agency for the Evaluation of Medicinal Products guidelines, while exhibiting favorable bleeding and weight profiles for at least 1 year.” |
| Meuwissen JHJM, L. Beijers-De Bie T, Vihtamaki R, Tuimala N, Siseles C, Magaril HS, Houben PWH, Murga C, Spielmann D, De Villiers TJ. | N/A |
| **Message #23**  Treatment with Premarin/trimegestone* has been shown to have a positive effect on the quality of life of postmenopausal women as evidenced by, alleviation of hot flush, acceptable amenorrhea levels, improvements in sleep patterns, no negative influence on mood or cognition. | |
| **Article** | **Message As Published** |
| Pornel B, Spielmann D. | “In summary, this study established the efficacy of the sequential 1mg 17ß-E_2_/0.25 mg TMG regimen in the significant reduction of vasomotor symptoms, psychofunctional disturbances and most sub-scales of the quality-of-life responses.” |
| Koninckx PR, Spielmann D. | N/A |
| Bouchard P, De Cicco-Nardone F, Spielmann D, Garcea N and the Trimegestone 301 Study Group. | “In conclusion, the findings of this large, randomized trial established the endometrial safety and good bleeding control of continuous combined 1 mg 17ß-E_2_/0.125 mg TMG throughout 2 years, confirming the potency and selectivity of TMG. Moreover, the accompanying paper shows that this regimen is efficient in the relief of vasomotor symptoms and psychofunctional disturbances associated with menopause.” |
| Gambacciani M, Spielmann D, Genazzani AR. | “Thus, an HRT combining estradiol with TMG should prevent endometrial hyperplasia while causing minimal interference with the beneficial effects of estradiol.”  “Overall, this study has demonstrated that the continuous combined regimen of 1 mg 17ß-E_2_/0.125 mg TMG is effective in relieving menopausal symptoms and improving quality of life in postmenopausal women.” |
| Meuwissen JHJM, L. Beijers-De Bie T, Vihtamaki R, Tuimala N, Siseles C, Magaril HS, Houben PWH, Murga C, Spielmann D, De Villiers TJ. | N/A |

**Reference:**

1. SONKJ031-001695 at SONKJO31-001720. (Publication Plan 2002 Premarin/Trimegestone HRT Version 1, July 19, 2002. Table 6. Communication Message analysis) [Internet]. San Francisco: Drug Industry Document Archive [cited 2009 Aug 21]. Available from: <http://dida.library.ucsf.edu/tid/wpc37b10>.

**Footnotes:**

*Three medical education companies were involved with trimegestone publications; eventually DesignWrite took over all trimegestone manuscripts from Parthenon and OCC North America. The planned marketing messages in this table were from an OCC document for promoting Premarin/trimegestone, but the messages remained similar for the estradiol/trimegestone publications.

**Documentation of ghostwriting for the above articles****

**a**: This article is listed as PS(1) on documents. Draft, with author tbd OLIVS019-002736, Winneker Depos., Exh. 61; Trimegestone: Publication and Abstract Tracking Report CONTA213-007655 at CONTA213-007657, Winneker Depos., Exh. 62; DESIGN045674, Winneker Depos., Exh. 88; WINNR201-013989 at WINNR201-013993, Winneker Depos., Exh. 64; WINNR401-004412 at WINNR401-004413 Winneker Depos., Exh. 63; CONTA213-016516 at CONTA213-016519 Winneker Depos., Exh. 66; CONSG204-024407, Winneker Depos., Exh. 65; CONTA213-028306 at CONTA213-028309, Winneker Depos., Exh. 67; OLIVS019-001933 at OLIVS019-001934, Winneker Depos., Exh. 69.

**b:** This article is listed as PS(2) on documents. OLIVS021-020865 at OLIVS021-020866, Winneker Depos., Exh. 72; CONTA025-023028, Winneker Depos., Exh. 3; OLIVS019-002754, Winneker Depos., Exh. 74; OLIVS019-002755, Winneker Depos., Exh. 75; WINNR201-013376, Winneker Depos., Exh. 76; WINNR201-013376 Winneker Depos., Exh. 76; DWRITE067378 at DWRITE067379, Winneker Depos., Exh. 77; CONTA213-008424 at CONTA213-008426, Winneker Depos., Exh. 79; WINNR201-013976 at WINNR201-013978, Winneker Depos., Exh. 80; CONTA213-026972, Winneker Depos., Exh. 82; OLIVS019-001933 at OLIVS019-001934, Winneker Depos., Exh.83.

**c:** This article is listed as PC(2) on documents. OLIVS019-001933; DESIGN046551; OLIVS019-019363, Winneker Depos., Exh. 125; Drafts: CONSG204-010720, DESIGN046016, CONSG204-010720; OLIVS019-002848, Winneker Depos., Exh. 126; OLIVS019-002849, Winneker Depos., Exh. 127; DESIGN046016 at DESIGN046017 Winneker Depos., Exh. 128; CONSG024-010712, Winneker Depos., Exh. 129; CONSG204-010713, Winneker Depos., Exh. 130;

DESIGN046551, Winneker Depos., Exh. 131; DESIGN046744; OLIVS019-001933.

**d:** This article is listed as PC(1) on documents. OLIVS019-019363; OLIVS020-007891; OLIVS019-002594, Winneker Depos., Exh. 85; OLIVS019-002593, Winneker Depos., Exh. 84; CONTA204-001232, Winneker Depos., Exh. 86; CONTA204-001257, Winneker Depos., Exh. 87; DESIGN046049, Winneker Depos., Exh. 89; DESIGN045674, Winneker Depos., Exh. 88; DESIGN057274, Winneker Depos., Exh. 90; OLIVS019-001933, Winneker Depos., Exh. 94.

**e**: This article is listed as P3(6)on documents. OLIVS019-024554 at OLIVS019-024555 and OLIVS019-024562; OLIVS019-001928, Winneker Depos., Exh. 56; WINNR201-007871 at WINNR201-007872; February 20, 2002 Progress Report Trimegestone 2mg CONTA025-023442.

** All documentation of ghostwriting is taken from Szaller J. Wyeth’s hormone therapies & ghostwritten medical literature (unpublished manuscript), with permission.
