# Supplementary material for: The Haunting of Medical Journals: How Ghostwriting Sold “HRT”
Source: PLoS Med. 2010 Sep 7;7(9):e1000335. doi: 10.1371/journal.pmed.1000335 (PMC2935455; doi:10.1371/journal.pmed.1000335)
Supplement: Table S2 — Examples of ghostwritten reviews and commentaries. (0.07 MB DOC) [file pmed.1000335.s002.doc]

**Table S2.** Examples of ghostwritten reviews and commentaries

| **Mitigating Perceived Risks of Breast Cancer** | |
| --- | --- |
| **Article** | **Documentation of Ghostwriting** |
| Creasman WT. Is There an Association between Hormone Replacement Therapy and Breast Cancer?  J Women’s Health 1998; 7(10). | Mittleman M137, DWRITE 068302 and DWRITE066231, Mittleman M21, DWRITE 003394, Mittleman M137, DWRITE 068302, DWRITE06776., Cobb Depos. 339:1-16, June 23, 2006., 1999 Madira Form letter, 2003 Magic form letters |
| Nachtigall LE. Sex Hormone-Binding Globulin and Breast Cancer Risk  Primary Care Update for Ob/Gyns 1999; 6 (2):39-45. | DWRITE068299 at DWRITE068300, DWRITE068299 at DWRITE068300, DWRITE068886, DWRITE067402, DWRITE067403, DWRITE067405, DWRITE068884, DWRITE068885, DWRITE068301 at 068302, Note: Acknowledges “editorial assistance of Ann Contijoch” |
| Eden J. Progestins and breast cancer. Am J Obstet Gynecol. 2003  May;188(5):1123-31. | CONTA025-03053, DESIGN174104, DESIGN018026, DESIGN018027, DWRITE066935, DWRITE003353, DWRITE066449, DWRITE003258, MARTN010-010296, SOLOJ027-048916, DWRITE066322, DESIGN134214 at DESIGN132215, DESIGN018080, DESIGN018170 and DESIGN018239, DESIGN136931, WRITE016984, DWRITE016989 at DWRITE016999, Note: Acknowledges “Karen D. Mittleman, Ph.D., and Stephen Parker, ELS, for their editorial assistance” |
| Cefalu T. The Use of Hormone Replacement Therapy in Postmenopausal Women with Type 2 Diabetes. J Women’s Health 2001; 10 (3):241-255 | DWRITE067403, DWRITE068308, DESIGN146717, DESIGN146698, Mittleman Depos. at 236, DWRITE068302, SOLOJ028-016914, DWRITE068302, DWRITE079093, DWRITE079094, DWRITE079095 and DWRITE079096 |
| **Promoting Unproven, Off-Label Uses** | |
| Fillit, M. The Role of Hormone Replacement Therapy in the Prevention of Alzheimer Disease. Arch Intern Med. 2002;162(17):1934-42. | Mittleman Depos. at 232, DWRITE066503, DWRITE003361, CONTA025-030538,  DESIGN174980, DESIGN174979, DESIGN174989, DELCA033-028519,  DUROJ023-001774 at DUROJ023-001775, COBBP010-000425, SOLOJ027-048916, DWRITE066543, DWRITE069866, CONTA025-019965 |
| Birge SJ. Practical Strategies for the Diagnosis and Treatment of Alzheimer’s Disease. Clinical Geriatrics 1999 7(4):56-74. | DWRITE067403, DWRITE068925, DWRITE067403, DWRITE068307, DWRITE068301, DWRITE068319, DWRITE067767 and DWRITE067797, DWRITE068308, DWRITE003381, DWRITE067767 |
| Shulman L. Is there a Connection Between Estrogen and Parkinson’s Disease? Parkinsonism Relat Disord. 2002;8(5): 289-95 | DWRITE068301 at DWRITE068303, DWRITE065923 at DWRITE 065925, DWRITE068135, Mittleman Depos. 232, DWRITE068135, DWRITE068135 at DESIGN068302, DWRITE068151, DWRITE068301 at DWRITE068303; and DWRITE068322 |
| Sherwin BB. Mild Cognitive Impairment: Potential Pharmacological Treatment Options. J Am Geriatr Soc. 2000;48(4):431-41. | DWRITE068301, DWRITE067409, DWRITE071026.DWRITE071097, DWRITE071098, Draft: DWRITE071052, Mittleman Depos. at 232, DWRITE068320,  DWRITE068142 |
| Brincat M, Baron Y, Galea R. Estrogens and the Skin. Climacteric 2005;8(2):110-23. | DWRITE067733, WEBEM015-022493 and DWRITE074105, DWRITE074046, DWRITE067733, DWRITE067294, Drafts: DWRITE074019, DWRITE074162 and DWRITE074047, A Strategic Publication Plan Tracking Report stated: “Review paper to include wound healing. “DWRITE067733 |
| Snow KK, Seddon JM. Age-Related Eye Diseases: Impact of Hormone Replacement Therapy and Other Risk Factors. Int J Fertil Womens Med. 2000 Sep-Oct;45(5):301-13 | DWRITE068302, Draft: DWRITE070295, DWRITE068322, DWRITE067768 |
| Freedman, MA. Quality of Life and Menopause: The Role of Estrogen.  J Women’s Health 2002;11(8):703-718. | DWRITE065923 at DWRITE 065925, DWRITE066320, Mittleman Depos. at 234,  DWRITE003374, DWRITE003249, DWRITE003273, DWRITE003250,  DWRITE066936, Drafts: WEBEM015-022424 and DWRITE071266, DWRITE066335, DWRITE066311, DWRITE 066506, DWRITE 066915, DWRITE 066916 |
| Bachman G, Leiblum S. The Impact of Hormones on Menopausal Sexuality: a Literature Review. Menopause 2004;11 (1): 120-130. | OLIVS019-025416 at OLIVS019-025422, DESIGN177338 at DESIGN177339,  DWRITE177342, DWRITE003333, Drafts of the outline: DWRITE074312 and DWRITE074337, DWRITE0067757 at DWRITE06775, Manuscript Drafts: DWRITE074346 and DWRITE074416, STRIS016-031138, DUROJ046-013385,  DWRITE075253, DWRITE074324, DESIGN024396, DESIGN024724, DESIGN024526, Note: Original title Menopause, Sexuality and HRT] [89] |
| **Competitive Messaging** | |
| Gallagher JC. Role of Estrogens in the Management of Postmenopausal Bone Loss. Rheum Dis Clin North Am. 2001;27(1):143-62. | DWRITE068317, DWRITE068319, DWRITE068301, DWRITE070957,  Note: Acknowledges “the contribution of Stephen M. Parker…” |
| Mosca L. The Role of Hormone Replacement Therapy in the Prevention of Postmenopausal Heart Disease, Arch Intern Med. 2000 Aug 14-28;160(15):2263-72. | DWRITE 068301, DWRITE 068320, DWRITE 068310, DWRITE003278, Note: Paper referred to as SERMS #4 in some documents |
| Warren M. A Comparative Review of the Risks and Benefits of Hormone Replacement Therapy Regimens.  Am J Obstet Gynecol. 2004 Apr;190(4):1141-67 | DWRITE072376, DWRITE072376, DWRITE072375, DWRITE072140, DWRITE072141, DWRITE066204 through DWRITE066208, Warren Depos. 253:1-24-254:1-24. Note: acknowledges Mittleman, Marren and Sendi. Note: article originally titled “A Comparative Review of the Risks and benefits of Hormone Replacement Therapy Regimens: How Generalizable Are They?” See DWRITE067763 |
| Curtis M. Selective Estrogen Receptor Modulators: A Controversial Approach for Managing Postmenopausal Health. J Women’s Health 1999; 8 (3) : 321-33 | DWRITE067407, DWRITE068811, DWRTIE068810, DWRITE003397,  DWRITE068326, DWRITE068319, DWRITE068326, Draft: DWRITE068843,  Note: Acknowledges “editorial assistance of Karen Mittleman, PhD, is greatly appreciated” |
| Curtis MG. Comparative Tolerability of First-Generation Selective Estrogen Receptor Modulators in Breast Cancer Treatment and Prevention.  Drug Safety 2001;24(14):1039-53 | DWRITE067407, DWRITE068811, DWRITE068810, DWRITE003397,  DWRITE068326, DWRITE068319, DWRITE068326, Draft: DWRITE068843,  Note: acknowledges “editorial assistance of Karen Mittleman, PhD, is greatly appreciated” |
| Bachmann GA. Menopausal Vasomotor Symptoms: a Review of Causes, Effects and evidence-Based Treatment, J Reprod Med. 2005 Mar;50(3):155-65. | Mittleman Depos. at 234, DWRITE003370, DWRITE067785, DWRITE031221, DWRITE031237, DWRITE066930, DWRITE066319, DWRITE066911 and DWRITE067764, DUROJ023-001712, Note: Acknowledges “the editorial assistance of Karen Mittleman, Ph.D., is greatly appreciated.” |
| Ansbacher R. The Pharmacokinetics and Efficacy of Different Estrogens are Not Equivalent. Am J Obstet Gynecol. 2001 Feb;184(3):255-63 | DWRITE003280, DWRITE068304, DWRITE003280, DWRITE068324, DWRITE060374, CONKJ018-004269 at CONKJ018-004270, DWRITE068324,  DESIGN176484, DWRITE068324, DESIGN176484, DWRITE068324,  DWRITE068324, DWRITE073454, DWRITE073455, DESIGN144069,  MARTN010-005742 at MARTN010-005743, Note: Article titled “All Estrogens are Not the Same for Postmenopausal Health” in some documents |
| No author listed. Generic and Therapeutic Substitution. National Pharmacy Compliance News 2000;4^th^ quarter:2-3. | DWRITE067467, DWRITE067100, DWRITE067120, Note: Author listed as Maddox R. in some documents |
| **Defending Cardiovascular Benefits** | |
| Mosca L. Hormone Replacement Therapy in the Prevention and Treatment of Atherosclerosis. Curr Atherosclerosis Reports 2000 Jul;2(4):297-302. | DWRITE 066432 at DWRITE066433, DWRITE 173881, DWRITE 069797,  DWRITE069748, DWRITE 069763, DWRITE069778, DWRITE069749,  DWRITE069746, DWRITE 067786, DWRITE 066519 |
| Rackley CE. New clinical markers predictive of cardiovascular disease: the role of inflammatory mediators. Cardiol Rev. 2004;12(3):151-7. | Mittleman Depos. 239:8-20, DWRITE067705, DELCA033-028519) DWRITE,  DELCA033-028521 at DELCA033-028522, DESIGN174518, COBBP010-000425, DWRITE067786, DESIGN174631, DWRITE003355, DWRITE069927, DWRITE069930, DWRITE066521, WEBEM015-012037DWRITE066922,  DWRITE003261, DWRITE067705, Drafts: DWRITE069935 and DWRITE069962,  DWRITE069927, DWRITE069930, DWRITE066935, DWRITE066936, DWRITE066937, Note: Acknowledges “Carolyn J. Smith, for assistance with research and Stephen M. Parker, ELS, for editorial assistance “ |
| Koh KK. Can a Healthy Endothelium Influence the Cardiovascular Effects of Hormone Replacement Therapy? Int J Cardiol. 2003;87(1):1-8 | CONTA023-008599, CONTA025-029754 at CONTA025-029755, CONTA025-029754 at CONTA025-029755, Mittleman Depos. at 233, DWRITE003343,  DWRITE066496, CONTA213-003673, CONTA025-029754, COBBP010-000425.  DWRITE066498, DESIGN175720 at DESIGN175721 and DESIGN175722  Note: acknowledges“…critical and devoted review [of] Richard O. Cannon III, M.D… [and] “the editorial assistance of Karen Mittleman, PhD.” |
| **Positioning Low-dose Therapy** | |
| Lobo R, Whitehead M. Is Low-Dose Hormone Replacement Therapy for Postmenopausal Women Efficacious and Desirable? Climacteric. 2001 Jun;4(2):110-9. | DWRITE073011, DWRITE73106, DWRITE072945, Note: Two years after publication, DesignWrite hired a writer “to update the Lobo and Whitehead review paper…”Mittleman Depos. at 238. The assignment form had “points to include” DWRITE072956 and DesignWrite supplied the references DWRITE072956 at DWRITE072958. It is unknown whether the updated review was published. |
| Maddox RW. The Efficacy and Safety of Low-dose Hormone Therapy. US Pharmacist 2004 (June). | DWRITE067467, DWRITE067100, DWRITE067120 |

* All documentation of ghostwriting is taken from Szaller J. Wyeth’s hormone therapies & ghostwritten medical literature (unpublished manuscript), with permission.
